# Caring for a Relative With Dementia in Long-Term Care During the COVID-19 Pandemic: A Prospective Longitudinal Study

**DOI:** 10.1093/geroni/igad034

**Published:** 2023-04-17

**Authors:** Lauren L Mitchell, Brenna Horn, Henry Stabler, Robyn W Birkeland, Colleen M Peterson, Elle A Albers, Joseph E Gaugler

**Affiliations:** Department of Psychology & Neuroscience, Emmanuel College, Boston, Massachusetts, USA; School of Public Health, University of Minnesota, Minneapolis, Minnesota, USA; School of Public Health, University of Minnesota, Minneapolis, Minnesota, USA; School of Public Health, University of Minnesota, Minneapolis, Minnesota, USA; Transportation Research Institute, University of Michigan, Ann Arbor, Michigan, USA; School of Public Health, University of Minnesota, Minneapolis, Minnesota, USA; School of Public Health, University of Minnesota, Minneapolis, Minnesota, USA

**Keywords:** COVID, 19, Dementia caregiving, Residential long, term care

## Abstract

**Background and Objectives:**

The coronavirus disease 2019 (COVID-19) pandemic introduced unprecedented threats and disruptions for caregivers of people with dementia living in residential long-term care (LTC) facilities. Qualitative and cross-sectional studies have reported substantial negative effects of the pandemic on dementia caregivers’ well-being, but little to no prospective research has examined the impact of COVID-19 on caregiver well-being using pre-pandemic assessments. The present study used longitudinal data from an ongoing randomized controlled trial of a psychosocial intervention to support family caregivers whose relatives had entered LTC.

**Research Design and Methods:**

Data collection began in 2016 and continued through 2021. Caregivers (*N* = 132) completed up to 7 assessments measuring their depressive symptoms, self-efficacy, and burden.

**Results:**

Latent growth curve models testing preregistered hypotheses revealed no significant effects of the pandemic on caregiver outcomes on average, though caregivers varied in terms of individual intercepts and slopes. Furthermore, factors such as caregiver–care recipient relationship closeness, care recipient’s COVID-19 infection status, and caregivers’ ratings of LTC facilities’ COVID-19 policies did not significantly moderate trajectories of well-being.

**Discussion and Implications:**

Findings highlight the heterogeneity of caregivers’ experiences during the pandemic, and suggest caution when interpreting cross-sectional findings on the impacts of the COVID-19 pandemic on caregiver well-being and distress.

**Translational Significance:** Family caregivers of persons with dementia living in residential long-term care (RLTC) faced many challenges during the coronavirus disease 2019 pandemic, including an unforeseen, lengthy period of prohibited visits accentuated with an uncertain resumption date. The effects of these added stressors on their well-being were heterogeneous. Many caregivers experienced surprisingly stable well-being across the pandemic. However, caregivers whose relatives’ RLTC quality declined during the pandemic were more likely to experience declines in well-being. These findings suggest that caregivers of relatives in RLTC whose care provision is more negatively impacted by unanticipated disruptions like a pandemic may benefit from additional support.

The coronavirus disease 2019 (COVID-19) pandemic has caused unprecedented challenges for chronically disabled individuals, and this has been especially true for those with dementia and their family caregivers. Older adults are at high risk for both chronic neurodegenerative conditions like dementia and poor COVID-19-related outcomes, and individuals with dementia have comprised a high proportion of COVID-19 deaths (Docherty et al., 2020; Nanda, Vura, & Gravenstein, 2020). Many older adults with dementia reside in long-term care (LTC) settings, which have been a source of significant COVID-19 transmission and whose residents have disproportionately died from COVID-19 (McMichael et al., 2020). Like many health care settings during the pandemic, LTC facilities frequently had to change their operations by instituting policies that limited human contact in order to mitigate the risk of infection. Restrictions included limiting or banning visitation from families of residents, which likely had negative implications for the health and well-being of residents and may have caused undue stress and worry for families (Mitchell et al., 2021). The pandemic also made caring for residents more difficult for LTC staff (White et al., 2021).

Understanding the effects that COVID-19 had on family caregivers’ mental health and well-being is important for planning care and services, both for individuals with dementia and their family caregivers. Family caregivers of people with dementia living in LTC settings often augment care provided by staff through socioemotional support, behavioral redirection, and assisting with activities of daily living such as eating (Baumbusch & Phinney, 2014; Gaugler, 2005). Although residential LTC placement may improve mental health for family caregivers over time, the transition can be a stressful process, especially for spouses (Afram et al., 2015; Gaugler, 2009; Gaugler et al., 2010; Graneheim et al., 2014; Matsuda et al., 1997; Schulz et al., 2004; Whitlatch et al., 2001). Studies have examined the impact of other transitions on caregivers for people with dementia living in LTC such as hospital transfers (Abrahamson et al., 2016) or the transition to end-of-life care (Williams et al., 2012). Through analyzing the impact of the COVID-19 pandemic on caregivers, we can better plan supports for future pandemics or other significant disruptions.

It is unclear what effects the COVID-19 pandemic has had on LTC caregivers’ well-being. Many studies have explored the impact of the COVID-19 pandemic on family caregivers of community-dwelling care recipients. Results have suggested high or increased levels of depression (Altieri & Santangelo, 2021), stress (Sheth et al., 2021), burden (Savla et al., 2020), anxiety (Beach et al., 2021; Hwang et al., 2021), and decreased social participation (Beach et al., 2021). One of the few studies that specifically investigated family caregivers of people with dementia living in LTC settings noted increased social isolation, strain, and reduced quality of life (Hindmarch et al., 2021). An important limitation of this work is that it has mostly relied on cross-sectional designs. Researchers have provided snapshots of mental health indicators (Beach et al., 2021; Hwang et al., 2021; Savla et al., 2020), compared cross-sectional samples (Sheth et al., 2021), and asked respondents to compare their mental health at present to prior to the pandemic (Altieri & Santangelo, 2021). The present study is one of the first prospective longitudinal studies of caregiver well-being prior to and during the pandemic. We were afforded the opportunity to study caregiver well-being prospectively by extending an ongoing longitudinal study that had begun prior to the COVID-19 pandemic.

The parent study, the Residential Care Transition Module (RCTM), began in late 2016 (Gaugler et al., 2020). The randomized controlled trial evaluated a six-session coaching program for family caregivers who had admitted a relative with memory loss to residential LTC. Coaching sessions focused on caregivers’ well-being and adjustment to their care recipient living in residential LTC. The study investigated the primary outcomes of family members’ subjective stress and emotional health as well as secondary role strains and residential care stress. As the RCTM study had begun prior to the onset of COVID-19 and the intervention and data collection continued without pause, the collection of prospective longitudinal data was possible. We were also able to augment the parent study surveys with additional items to assess caregivers’ pandemic-related experiences in order to better understand how these experiences influenced caregivers’ well-being during the pandemic.

The purpose of the present study was to investigate prospective changes in the well-being of family members caring for a relative with dementia living in LTC before and during the COVID-19 pandemic. Three research questions guided the current analysis. First, among family caregivers of people with dementia living in residential LTC, how have caregiver stress and mental health changed after the onset of the COVID-19 pandemic? We hypothesized that caregivers would experience decreases in self-efficacy and increases in caregiver burden and depressive symptoms when comparing post-pandemic to pre-pandemic assessments.

Second, how were COVID-19 pandemic effects on caregiver well-being moderated by the type and quality of the relationship between the caregiver and care recipient, the care recipient’s level of cognitive impairment, perceptions of LTC staff support, and ratings of the LTC facility’s handling of the pandemic? We selected these moderators in the early stages of the pandemic, and thus our selection was guided by early emerging literature and news, as well as discussions with LTC staff, healthcare practitioners, and caregivers (e.g., Barnett & Grabowski, 2020; Stockman et al., 2020; University of Minnesota, 2020).

We expected the sharpest decreases in well-being in spouses, in caregivers who reported a closer relationship with their care recipient prior to the pandemic onset, whose care recipient had greater levels of cognitive impairment at the start of the pandemic, who perceived lower levels of LTC staff support prior to the pandemic onset, and who rated their facility’s handling of the COVID-19 pandemic more negatively. Although closer relationships are associated with better adjustment to residential care (Whitlatch et al., 2001), in the particular context of COVID-19, we expected that forced separation would be more disruptive for closely bonded and highly interdependent caregiver–care recipient dyads than for those whose relationships were more distant. Care recipients with more severe cognitive impairment may have greater difficulty understanding the reasons for social distancing and other safety protocols, and we expected this would contribute to greater caregiver distress (Brown et al., 2020). Finally, positive, trusting relationships between caregivers and LTC staff have been identified as a crucial factor in supporting family caregiver involvement in LTC, and ultimately promoting residents’ quality of life (Hoek et al., 2021; Roberts & Ishler, 2018). Thus, we expected that caregivers who perceived a high level of staff support and who felt positively about their facility’s pandemic response may have been buffered against pandemic-related stress (see also Mitchell et al., 2021).

Lastly, does the RCTM intervention protect against the negative consequences of the COVID-19 pandemic? We predicted that participants in the treatment group would experience better well-being and lower stress after the pandemic onset than participants in the control group. These research questions and hypotheses, along with our analysis plan, were preregistered on the Open Science Framework (https://osf.io/dfsg7).

## Method

### Participants

Recruitment for the parent RCTM study was conducted via newspaper advertisements, outreach to LeadingAge members, and through the (blinded for review) Caregiver Registry (detailed recruitment information can be found in Gaugler et al., 2020). Eligibility for the study consisted of being the most involved with, or sharing the role equally of, providing care to a relative living in residential care (e.g., nursing home, assisted living facility) with a diagnosis of Alzheimer’s Disease and Alzheimer’s Disease Related Dementias (AD/ADRD), being 21 years old or older, and English speaking.

Among the *N* = 240 participants in the parent study, *N* = 132 provided at least one wave of caregiver outcome data after the onset of the COVID-19 pandemic and were included in the present analyses. The vast majority of participants in this subset were either providing care for a parent (61%) or their spouse (29%). Most caregivers were female (86%), White (97%), and highly educated (77% had a Bachelor’s degree or higher). Care recipients were, on average, 82 years old. Most care recipients were female (66%) and White (97%). See Table 1 for further demographic information about caregivers and care recipients.

**Table 1.** Demographic Characteristics of Treatment/ Control Caregivers and Care Recipients.

Variable	Mean (*SD*)	*n* (%)
Caregiver characteristics		
Age, in years	62.2 (10.3)	
Female		113 (85.6)
White, non-Hispanic		128 (97.0)
Bachelor’s degree or higher		102 (77.3)
Married and/or living with partner		107 (81.1)
Number of children	2.0 (1.5)	
Relationship to care recipient		
Spouse/partner		38 (28.8)
Daughter or son		81 (61.4)
Other		13 (9.8)
Care recipient characteristics		
Age, in years	82.1 (7.8)	
Female		87 (65.9)
White, non-Hispanic		128 (97.0)
Bachelor’s degree or higher		54 (41.0)
Medicaid recipient		39 (29.5)
Months spent in long-term care (reported at baseline)	14.3 (17.1)	

*Note*: *SD* = standard deviation.

### Procedure

The parent study evaluated the semistructured psychosocial RCTM intervention through a randomized controlled trial, with 120 participants assigned to a usual care condition and 120 participants receiving the intervention. This coaching program consisted of six 1- to 2-hr sessions (individual and/or family sessions) delivered over the course of four months to family caregivers who had admitted a relative with memory loss to residential LTC. Session topics included psychoeducation about dementia, stress management, communication and conflict resolution, guilt, and grief. Participants could also request ad hoc sessions at any time during the 12-month period in which they were enrolled (see Gaugler et al., 2020, for detailed information on the intervention). Participants were scheduled for a series of surveys at baseline, 4 months, 8 months, and 12 months.

**Figure 1.**
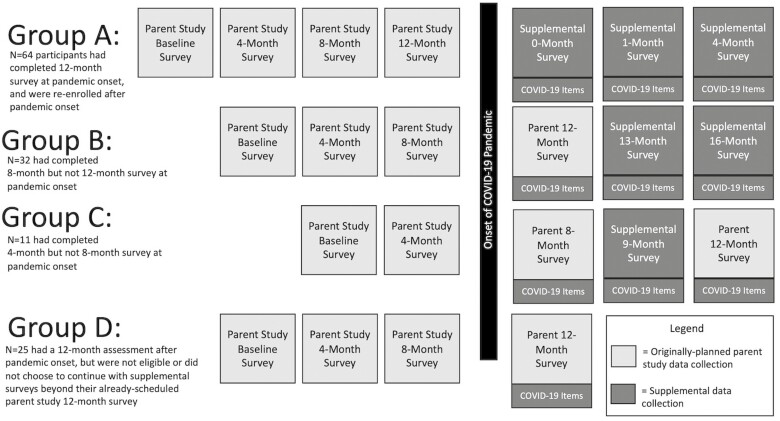
*Survey administration timeline.* COVID-19 = coronavirus disease 2019.

All participants completed their baseline survey for the RCTM parent study before the start of the pandemic. To extend data collection further into the pandemic period, we invited participants to complete up to three supplemental surveys, spaced 1–3 months apart, so that each participant had an opportunity to take part in at least three assessments after the pandemic began (see Figure 1). Supplemental surveys included the same measures as parent study surveys, with additional items evaluating caregivers’ experiences related to the COVID-19 pandemic (e.g., whether their relative was infected with COVID-19; satisfaction with the LTC’s COVID-19 policies). Participants who had already completed their 12-month survey for the parent study before the pandemic were contacted and invited to re-enroll in the study if not already bereaved (given that participants whose relative had already passed away at the time of our re-recruitment efforts had likely never experienced caring for a relative in LTC during pandemic conditions, we did not re-engage these participants in the study). Participants who remained currently active in the parent study at the time of the pandemic onset were invited to extend their participation beyond their planned 12-month survey. Survey distribution and data management procedures from the parent RCTM study were continued (Gaugler et al., 2020). Blinded study staff distributed online or hard copy surveys and corresponded with participants to facilitate the completion of surveys. Participants were compensated $25 for each returned survey. The study was approved by the University of (blinded for review) IRB (Protocol #1511S80406).

Because participants were at different stages in the parent study timeline when the COVID-19 pandemic began, individual times of assessment varied with respect to the pandemic onset (see Figure 1). To accommodate these variations, individually varying times of assessment were incorporated into longitudinal models, as described in the Analysis Plan subsequently.

### Measures

#### Caregiver depressive symptoms

Caregiver depression was assessed with the 20-item Center for Epidemiological Studies—Depression scale (CES-D), which assesses symptoms such as depressed mood, feelings of guilt and worthlessness, feelings of helplessness and hopelessness, psychomotor retardation, loss of appetite, and sleep disturbance (Radloff, 1977). The CES-D is a validated measure for depression in the general population as well as dementia family caregivers (Ying et al., 2019). Respondents indicate how frequently each of the 20 symptoms occurred in the past week scoring from 0 (rarely or none of the time/less than 1 day) to 3 (most of the time/5–7 days). Cronbach’s alpha ranged from 0.81 to 0.94 across waves.

#### Caregiver self-efficacy

The family caregiver’s self-efficacy was assessed using eight items adapted from Fortinsky et al.’s (2002) self-efficacy instrument for dementia family caregivers. Participants were asked to rate how confident they felt at the time on a scale from 1 (very unconfident) to 5 (very confident) in various caregiving-related situations, such as “handle any problems your relative may have” and “find organizations or agencies in the community that provide services to help you and/or your relative.” Alpha ranged from 0.83 to 0.94 across waves.

#### Caregiver burden

Caregiver burden was assessed using seven items from the 22-item Zarit Burden Inventory or Interview (ZBI; Zarit, 1985), which was developed to measure the burden for this population. Specifically, this seven-item version has been used with dementia family caregivers after their care recipient has been admitted to a nursing home (Gaugler, 2009; Gaugler et al., 2010). Participants were asked on a scale of 0 (never) to 4 (nearly always), how frequently they experienced various indicators of burden such as feeling “stressed between caring for [their] relative and trying to meet other responsibilities for [their] family or work.” Alpha ranged from 0.86 to 0.91.

#### Care recipient’s dementia severity

The care recipient’s cognitive impairment was measured by the Memory Impairment Scale and the Revised Memory and Behavior Problems Checklist (RMBPC). The Memory Impairment scale was developed to assess the cognitive status of persons living with Alzheimer’s disease as reported by family caregivers (Pearlin et al., 1990). The scale contains eight items about the difficulty the care recipient experiences with certain cognitive tasks. For example, participants are asked, “how difficult is it for your relative to know what day of the week it is?” Response options are on a 5-point Likert scale from 0 (not difficult at all) to 4 (can’t do at all). Alpha ranged from 0.88 to 0.91 across waves.

In the RMBPC, family caregivers report on the frequency of 24 dementia-related behaviors in the past week and the impact of those behaviors on the caregiver (Teri et al., 1992). Sample items include, “asking the same question over and over” and “aggressive to others verbally.” For the frequency of behaviors (RMBPC-Frequency), response options span from 0 (never occurred) to 4 (daily or more often). For severity of behaviors, respondents are asked how much the behavior bothered or upset them (RMBPC-Severity) from 0 (not at all) to 4 (extremely).

#### Relationship type and closeness

Participants selected their relationship to the care recipient with six options: spouse or partner, daughter or son, daughter-in-law or son-in-law, sister or brother, friend, or other (with an open-ended field). The strength of the relationship between the caregiver and care recipient was measured with the Relationship Closeness Scale (RCS), a six-item instrument developed by Whitlach et al. (2001) as part of the Stress Process Model for Residential Care. Items include statements such as “my relative always made me feel like a special person.” Response options ranged from 1 (strongly disagree) to 5 (strongly agree). Alpha ranged from 0.89 to 0.95.

#### Support from LTC staff

The level of support caregivers felt they received from LTC staff was measured with five items also developed by Whitlach et al. (2001). For example, caregivers were asked how often direct care workers “reassure you that your relative’s behavior is not unusual” and “keep you informed about changes in your relative’s condition.” Response options include 3 (most of the time), 2 (some of the time), and 1 (hardly ever). Alpha ranged from 0.77 to 0.83 across waves.

#### LTC centers’ pandemic response

Caregiver perceptions of the LTC center’s COVID-19 response were assessed with five items informed by ongoing discussions of COVID-19 and LTC and literature available in the Spring of 2020 (University of Minnesota School of Public Health Center on Aging, 2020; Wang et al., 2020), as well as counseling session notes from the parent study. Participants were asked to rate on a scale from 1 (very unsatisfactory) to 5 (very satisfactory) the LTC center’s degree of preparedness, communication about COVID-19 policies to residents and families, effectiveness of preventive measures, efforts to keep residents engaged, and confidence in the center’s ability to prevent or slow the spread of COVID-19. Alpha ranged from 0.71 to 0.93 across study waves.

#### Overall rating of LTC quality

Participants were asked to rate the overall quality of their relative’s LTC facility using a single item: “Using any number from 0 to 10, where 0 is the worst care possible and 10 is the best care possible, what number would you use to rate the care at your relative’s facility?” We calculated the average score across all pre-pandemic waves, as well as the change in average score pre-pandemic to post-pandemic-onset.

#### Social support

Caregivers’ overall level of social support was assessed with the Socioemotional Support Scale (Schieman & Meersman, 2004), an eight-item item measure. A sample item is, “You have at least one friend or relative you can really confide in.” Participants rated items on a scale from 1 (strongly disagree) to 5 (strongly agree). Alpha ranged from 0.90 to 0.94.

#### Care recipient’s COVID-19 infection status

Caregivers were asked “Has your relative had COVID-19?” in post-pandemic-onset surveys. Participants could respond yes, no, not sure, or that their relative experienced cough, fever, or other flu-like symptoms but was not diagnosed or tested. Participants who responded “yes” at any point were coded as having experienced their care recipient being infected with COVID-19.

### Analysis Plan

We tested our primary hypotheses using latent growth curve models (LGM; Singer & Willett, 2003), creating separate models for each outcome. We used Mplus version 8 for LGM analyses (Muthén & Muthén, 1998-2017). For all models, individually varying times of assessment were used to account for participants’ variation in timing of assessments relative to the COVID-19 pandemic onset. Each assessment was assigned a time score, reflecting the number of days before or after the pandemic onset. Sample sizes approaching 100 or greater are considered adequate for latent growth curve modeling (Curran et al., 2010).

First, we fit unconditional growth curve models to determine functional form. We fit intercept-only, linear, and quadratic models and retained the model with the best-fit statistics. Lower Akaike Information Criterion (AIC) and Bayesian Information Criterion (BIC) values indicated a better fit (Hu & Bentler, 1999). Other fit statistics (e.g., chi-square, CFI, RMSEA) are not available when incorporating individually-varying times of assessment (Muthén & Muthén, 2008). Given this, we also examined the magnitude and significance of intercepts and variances within the unconditional models, and selected functional forms that balanced parsimony with fit.

After determining the functional form, we incorporated covariates into the model to address our research questions. We used a dummy variable to denote whether each assessment occurred before or after March 13, 2020 (0 = pre-pandemic onset, 1 = post-pandemic onset). This variable was entered as a time-varying predictor at each wave. Demographic variables that were significantly correlated with more than two waves were entered for each outcome as time-invariant predictors. Our primary moderators (cognitive impairment, relationship closeness, staff support, satisfaction with LTC’s COVID-19 strategy, and spousal relationship) were entered as time-invariant predictors to examine their direct effects. For covariates that were measured at multiple waves, we calculated a mean score across all available waves prior to the pandemic onset for each participant. We also entered time-varying covariates for the interaction between these moderators and pandemic onset, to examine whether any moderators intensified or dampened the effects of pandemic onset on outcomes. The parent study treatment group was similarly entered as a time-invariant predictor and the interaction between treatment group and pandemic onset was entered as a time-varying predictor. Bereavement was included as a predictor for depressive symptom models, but not for caregiver self-efficacy or caregiver burden because bereaved caregivers did not complete assessments of self-efficacy or burden. Household income, cognitive impairment, relationship closeness, staff support, and satisfaction with LTC’s COVID-19 strategy were centered to facilitate the interpretation of results. The Holm correction (Holm, 1979) was applied to the final models to control for family-wise error.

We also conducted exploratory analyses to examine the potential moderating effects of additional variables that were not part of our original hypotheses, but emerged as notable factors in caregivers’ COVID-19 experiences through our previous analysis of open-ended qualitative data from the present sample (Mitchell et al., 2021). These qualitative findings suggested that positive perceptions of LTC facilities and staff, including good communication with staff, were perceived as important buffers against COVID-19-related stress. In contrast, caregivers were negatively affected when they felt quality of care declined as a result of understaffing, and when infection prevention strategies were inconsistently enforced by LTC staff. Caregivers reported substantial worry about LTC residents being infected with COVID-19. They also identified social support as a key resource in helping them cope with the pandemic. We were able to relate these qualitative themes to variables that had been quantitatively assessed, and could thus be included as moderators in our models. These included overall rating of LTC facility, change in ratings of LTC from before pandemic onset to after pandemic onset, care recipient being infected with COVID-19, satisfaction with LTC’s communication around COVID-19, and social support. We took a similar approach to that described earlier for these additional moderators, incorporating them directly as time-invariant covariates, and in time-varying covariates reflecting the interaction between each moderator and pandemic onset.

## Results

### Preliminary Analyses

Correlations between descriptive statistics and outcomes are reported in Supplementary Tables 1–3. We compared the pre-pandemic characteristics of participants who had complete data for all seven possible waves of the study to those who had fewer than seven waves of data. Results of these tests are reported in Supplementary Table 4. Participants did not differ significantly on most characteristics with three exceptions: those with complete data had somewhat lower levels of relationship closeness, perceived slightly lower levels of staff support, and had somewhat lower self-efficacy scores before the onset of the COVID-19 pandemic compared to those with fewer than seven waves of data.

### Unconditional Models

For depressive symptoms, the linear unconditional model resulted in the lowest BIC value (see Table 2). The coefficient for quadratic slope in the quadratic unconditional model was also not statistically different from zero (*Q* = −0.009, *p* = .54) and variance in quadratic slope was minimal (Var = 0.007, *p* = .047). Thus, we selected the linear functional form for depressive symptoms. This model indicated that on average, participants scored low on depressive symptoms at baseline (*I* = 0.63, *p* < .001) and remained fairly stable across the study (*S* = −0.04, *p* = .09), but with significant variability in linear slopes (Var = 0.02, *p* = .007).

**Table 2.** Fit Indices for Latent Growth Curve Models

Model	AIC	BIC
DEP: Unconditional constant	794.41	820.28
DEP: Unconditional linear	762.64	797.14
DEP: Unconditional quadratic	756.41	802.41
DEP: Conditional model	696.77	813.67
ZBI: Unconditional constant	1193.12	1218.99
ZBI: Unconditional linear	1046.65	1081.15
ZBI: Unconditional quadratic	1050.40	1096.40
ZBI: Conditional model	970.77	1096.23
CSE: Unconditional constant	1391.56	1417.44
CSE: Unconditional linear	1334.62	1369.12
CSE: Unconditional quadratic	1338.91	1384.91
CSE: Conditional model	1187.74	1301.54

*Notes*: AIC = Akaike Information Criterion; BIC = Bayesian Information Criterion; CSE = caregiver self-efficacy; DEP = depressive symptoms; ZBI = Zarit Burden Inventory.

For caregiver self-efficacy, the unconditional linear model provided the lowest AIC and BIC values (see Table 2), and thus the linear functional form was selected. The unconditional model revealed that caregivers felt moderately confident in their caregiving skills on average at baseline (*I* = 3.64, *p* < .001) and increased slightly over the study period (*S* =0.09, *p* = .006).

For caregiver burden, the lowest AIC and BIC values were obtained from the unconditional linear model (see Table 2). Therefore, we used the linear functional form for caregiver burden. The unconditional model suggested that, on average, caregivers scored moderately on burden at baseline (*I* = 1.42, *p* < .001) and experienced slight declines in burden over the course of the study (*S* = −.24, *p* < .001).

### Conditional Growth Models

Pandemic onset was not significantly associated with depressive symptoms, self-efficacy, or burden (see Table 3). Furthermore, none of the key situational factors that we examined (i.e., cognitive impairment; relationship closeness; LTC staff support; rating of facility’s handling of COVID-19; spousal relationship to care recipient) significantly moderated the effect of pandemic onset on any of the outcomes. Prior to applying the Holm correction, one measure of dementia symptom severity (RMBPC-Severity) seemed to be associated with a more positive effect of pandemic onset on caregiver self-efficacy (*B* = 0.26, *p =* .004), but this effect was no longer significant after applying the correction. The RCTM intervention also did not significantly moderate the effects of pandemic onset on caregivers’ outcomes.

**Table 3.** Latent Growth Curve Models Predicting Depressive Symptoms, Caregiver Self-Efficacy, and Caregiver Burden

	Depressive symptoms		Self-efficacy		Burden	
	Coefficient	*SE*	Coefficient	*SE*	Coefficient	*SE*
Fixed effects						
For intercept						
Intercept	0.65^***^	0.12	3.70^***^	0.13	0.81^†^	0.28
Race/ethnicity: Non-White	0.59^***^	0.14	−0.53	0.33	—	—
Married	−0.14	0.14	0.09	0.13	−0.15	0.20
Caregiver household income	−0.04	0.02	0.03	0.03	—	—
Primary caregiver	—	—	—	—	0.29	0.16
Female	—	—	—	—	0.42^†^	0.17
Number of children	—	—	—	—	−0.08^†^	0.04
Medicaid	—	—	—	—	−.12	.12
Spouse	.14	.10	−.25	.15	.12	.17
Memory impairment	-0.02	0.06	−0.06	0.09	−0.14	0.10
RMBPC-frequency	0.15	0.08	−0.02	0.14	0.33^†^	0.13
RMBPC-severity	0.09	0.06	−0.24^†^	0.10	0.18	0.11
Relationship closeness	0.07	0.05	0.14^†^	0.07	0.10	0.09
Staff Support	−0.16	0.11	0.29^†^	0.13	−0.08	0.17
LTC COVID-19 strategy	−0.08	0.06	0.22^**^	0.07	−0.13	0.10
Treatment	0.08	0.09	−0.06	0.08	0.19	0.14
For linear slope						
Intercept	−0.09	0.05	0.22^†^	0.10	−0.08	0.09
Race/ethnicity: non-White	−0.20	0.13	−0.14	0.18	-	-
Married	0.05	0.05	−0.01	0.11	0.08	0.08
Caregiver household income	0.001	0.01	0.02	0.02	-	-
Primary caregiver	-	-	-	-	−0.04	0.05
Female	-	-	-	-	−0.21^**^	0.06
Number of children	-	-	-	-	−0.006	0.02
Medicaid	—	—	—	—	0.07	0.05
Spouse	−0.005	0.04	−0.09	0.08	−0.10	0.06
Memory impairment	−0.006	0.04	−0.03	0.05	−0.07^†^	0.03
RMBPC-frequency	0.03	0.04	0.11	0.07	0.11	0.06
RMBPC-severity	−0.04	0.03	−0.04	0.06	<0.001	0.05
Relationship closeness	−0.04	0.03	0.009	0.05	−0.04	0.05
Staff support	0.05	0.05	0.08	0.08	0.01	0.07
LTC COVID-19 Strategy	−0.01	0.02	0.04	0.03	0.01	0.03
Treatment	0.04	0.04	−0.21^***^	0.06	0.01	0.06
Fixed effects for time-varying covariates						
Pandemic onset	0.06	0.05	−0.03	0.07	−0.08	0.07
Spouse × PO	−0.08	0.07	−0.06	0.11	0.12	0.10
Memory Impairment × PO	0.01	0.06	0.01	0.08	−0.04	0.06
RMBPC-Frequency × PO	0.02	0.06	−0.17	0.12	−0.04	0.07
RMBPC-Severity × PO	0.006	0.04	0.26^†^	0.09	−0.04	0.05
Relationship Closeness × PO	−0.03	0.04	−0.02	0.07	−0.09	0.05
Staff Support × PO	−0.03	0.09	0.08	0.13	−0.14	0.14
Treatment × PO	−0.08	0.06	0.16	0.10	0.06	0.08
Bereavement	0.05	0.07	—	—	—	—
Random effects						
Intercept	0.12^***^	0.02	0.16^***^	0.05	0.34^***^	0.04
Linear Slope	0.01^†^	0.006	0.03^†^	0.01	0.01	0.01

*Notes*: COVID-19 = coronavirus disease 2019; LTC = long-term care; PO = pandemic onset; RMBPC = Revised Memory and Behavior Problems Checklist; *SE* = standard error. All coefficients are unstandardized. Models control for demographics that were correlated with outcomes.

^***^ *p* < .001.

^**^ *p* < .05 after Holm correction.

^†^ *p* < .05 before Holm correction.

Some notable effects that we did not hypothesize emerged. In particular, caregivers who rated the LTC’s strategy for addressing COVID-19 highly at baseline also scored significantly higher on self-efficacy at baseline (*B* = 0.22, *p* = .001). Furthermore, the RCTM intervention was associated with declines in caregiver self-efficacy over time, unrelated to COVID-19 onset (*B* = −0.21, *p* < .001).

We conducted exploratory analyses informed by our previous qualitative analysis of open-ended data from participants describing their experiences related to COVID-19. The moderators we explored included: overall LTC quality; change in LTC quality from pre- to post-pandemic onset; social support; whether the care recipient was infected with COVID-19; and ratings of LTC communication quality around COVID-19. None of these variables significantly moderated the effect of COVID-19 on caregiver outcomes (see Table 4). However, prior to applying the Holm correction, caregivers who experienced more positive changes in LTC quality after the start of the pandemic seemed to experience greater declines in burden after pandemic onset (*B* = −0.08, *p* = .02). This effect was not statistically significant after applying the correction.

**Table 4.** Latent Growth Curve Models Predicting Depressive Symptoms, Caregiver Self-Efficacy, and Caregiver Burden on Qualitative-Informed Moderators

	Depressive symptoms		Self-efficacy		Burden	
	Coefficient	*SE*	Coefficient	*SE*	Coefficient	*SE*
Fixed effects						
For intercept						
Intercept	0.74^***^	0.04	3.59^***^	0.11	1.02^**^	0.30
Race/Ethnicity: Non-White	0.42	0.22	−0.20	0.19	—	—
Married	−0.15	0.11	0.04	0.11	−0.03	0.19
Caregiver Household Income	−0.02	0.02	0.04	0.03	—	—
Primary Caregiver	—	—	—	—	0.25	0.14
Female	—	—	—	—	0.31	0.17
Number of children	—	—	—	—	−0.07	0.04
Medicaid	—	—	—	—	−0.27^†^	0.13
LTC Quality	−0.02	0.04	0.05	0.04	−0.11	0.06
Change in LTC Quality	0.01	0.03	0.02	0.03	0.001	0.04
Social Support	−0.29^**^	0.09	0.48^***^	0.12	−0.22	0.13
Care Recipient Infected	−0.08	0.08	0.05	0.10	−0.04	0.15
LTC Communication	−0.10	0.05	0.14	0.08	−0.08	0.09
For linear slope						
Intercept	−0.02	0.04	0.13	0.13	−0.06	0.12
Race/Ethnicity: Non−White	−0.13	0.09	−0.13	0.18	—	—
Married	0.02	0.04	−0.03	0.12	0.02	0.06
Caregiver Household Income	0.008	0.01	0.02	0.02	—	—
Primary Caregiver	—	—	—	—	−0.07	0.05
Female	—	—	—	—	−0.11	0.07
Number of children	—	—	—	—	0.005	0.01
Medicaid	—	—	—	—	0.04	0.05
LTC Quality	0.004	0.02	−0.02	0.03	−0.004	0.02
Change in LTC Quality	−0.04^†^	0.01	0.02	0.03	−0.04^†^	0.02
Social Support	−0.002	0.03	0.12	0.06	−0.04	0.05
Care Recipient Infected	−0.02	0.04	0.01	0.07	−0.03	0.05
LTC Communication	0.01	0.02	0.02	0.04	0.04	0.04
Fixed effects for time-varying covariates						
Pandemic Onset	0.02	0.03	0.06	0.06	0.01	0.05
Facility Quality x PO	0.007	0.02	0.06	0.04	−0.03	0.03
Change in Quality x PO	−0.03	0.02	−0.04	0.03	−0.08^†^	0.03
Social Support x PO	−0.01	0.05	−0.03	0.11	0.11	0.07
Care Recipient Infected x PO	0.03	0.06	−0.10	0.10	−0.04	0.08
LTC Communication x PO	−0.03	0.04	−0.10	0.10	0.08	0.05
Bereavement	0.01	0.06	—	—	—	—
*Random Effects*						
Intercept	0.12^***^	0.02	0.14^†^	0.05	0.36^***^	0.04
Linear Slope	0.009^†^	0.005	0.03^†^	0.01	0.01	0.008

*Notes*: LTC = long-term care; PO = pandemic onset; *SE* = standard error. All coefficients are unstandardized. Fixed effects represent the average trajectory across all participants, and random effects represent the variance of individual participants’ trajectories around the average trajectory. Models control for demographics that were correlated with outcomes.

^***^ *p* < .001.

^**^ *p* < .01.

^*^ *p* < .05 after Holm correction.

^†^ *p*< .05 before Holm correction.

## Discussion

Overall, caregivers seemed to improve over time in terms of depressive symptoms, self-efficacy, and burden, likely reflecting improvements in caregiver well-being after LTC placement (e.g., Gaugler, 2009; Gaugler et al., 2010; Matsuda et al., 1997). Although we expected caregivers’ well-being would worsen after the onset of the COVID-19 pandemic, we found no evidence of overall declines in well-being. However, most outcomes demonstrated significant variability in slopes, indicating that some caregivers experienced increases and others experienced declines in well-being over time. Qualitative research on caregiving during COVID-19 has revealed mixed effects of the pandemic, with some caregivers experiencing severe negative effects and others finding “silver linings” despite the various challenges (e.g., Lightfoot et al., 2021). Our own qualitative work with the current sample highlighted many stressors such as worry about care recipients’ isolation, potential infection, and negative end-of-life experiences in LTC settings during the pandemic (Mitchell et al., 2021). At the same time, some participants noted that lockdowns reduced guilt about visiting care recipients who no longer remembered or responded to them. Still others felt a moderate level of stress that was offset by a strong sense of trust in the LTC staff and high-quality care. Thus, the COVID-19 pandemic has had heterogeneous effects on well-being for caregivers of LTC residents with dementia.

We examined a wide range of possible moderators of caregiver well-being that might help explain which caregivers were at the highest risk of declines in well-being. The moderators we tested included factors related to the caregiver, the care recipient, and the LTC facility, yet these tests yielded no significant interactions. Two moderators were significantly associated with change in well-being before applying the Holm correction, although those effects were no longer significant after correction. Specifically, participants who reported that their care recipient experienced more severe behavioral symptoms of dementia seemed to experience steeper increases in self-efficacy after COVID-19 emerged. In addition, caregivers who reported a more positive change in LTC quality from pre- to post-pandemic onset exhibited steeper declines in burden after the start of the pandemic. Although we emphasize that these findings were no longer significant after correction, dementia severity and changes in LTC quality may represent fruitful directions to explore in future research to explain caregivers’ adaptation to the pandemic. Recently-published research also suggests that factors we were unable to rigorously explore, including larger facility bed size and location in an area with high COVID-19 infection prevalence, were associated with more COVID-19 cases and deaths within LTC facilities (Konetzka, White, Pralea, Grabowski, & Mor, 2021). Thus, LTC facility size and local COVID-19 prevalence may be worth investigating in future research on the effects of COVID-19 on caregiver well-being. Furthermore, the psychosocial support provided through the RCTM intervention did not seem to buffer the effects of COVID-19 on caregivers’ well-being. This finding is consistent with the primary intervention outcomes analyses reported by Gaugler et al. (2023). Although participants in the treatment group generally perceived the intervention as useful and beneficial, quantitative results suggested that the RCTM intervention had null and in some cases negative effects on caregivers’ well-being on average (see Gaugler et al., 2023, for a full discussion). For example, it may be that the intervention inadvertently led caregivers to feel dependent on the support provided by RCTM interventionists, which could explain the decline in self-efficacy we observed among treatment group participants.

Notably, our findings diverge from many cross-sectional and retrospective studies reporting the negative impacts of COVID-19 on caregiver well-being (e.g., Altieri & Santangelo, 2021; Beach et al., 2021; Hwang et al., 2021; Savla et al., 2020). The substantial negative impacts reported in these cross-sectional studies do not seem to align with the overall neutral effects that our prospective findings suggest. Such divergence between prospective and retrospective designs is exemplified in other areas of research, such as the literature on post-traumatic growth (e.g., Helgeson et al., 2006; Tedeschi & Calhoun, 2004). Much of the early literature on post-traumatic growth utilized retrospective measures asking participants to remember and compare their past self to their present self (Tedeschi & Calhoun, 1996), much like the majority of current research on COVID-19 and caregiver well-being. These retrospective studies frequently demonstrated substantial perceived benefits of traumatic events for character development and growth. However, longitudinal research comparing the same individuals before and after a traumatic event revealed little to no systematic change (Frazier et al., 2009). Prospective research is especially critical for accurately understanding the impacts of major, disruptive events. Although collecting pre- and post- data may be logistically impossible in many cases, ongoing studies such as the RCTM intervention provide unique opportunities to compare the same set of individuals before and after a major event like the COVID-19 pandemic onset.

Despite this important strength, the present study had notable limitations. First, the participants in this study were relatively highly educated and primarily White. Their privileges and resources may have protected them from many consequences of the pandemic or afforded them greater support in coping with stressors that ultimately protected their well-being. For example, nursing homes serving a greater proportion of Black and Latinx residents have experienced greater rates of COVID-19 infection (Gebeloff, 2021) and overall rates of infection and mortality due to COVID-19 are higher among Black and Latinx groups (Mackey et al., 2021). These risks introduced by the pandemic compounded existing racial and ethnic disparities in long-term services and supports (Shippee et al., 2020). Research with more representative samples, and especially research on populations that have been systematically excluded from resources and services to mitigate the effects of COVID-19, is critically needed. Second, we were limited to examining those well-being measures that were included in the parent study, and therefore missed constructs such as anxiety, which may be more severely impacted by an event like the COVID-19 pandemic. Finally, because data collection ended in the summer of 2021, we were unable to examine more long-term trajectories as families continue to adapt to the ongoing pandemic.

## Conclusion

Our findings highlight the heterogeneity of caregivers’ experiences and well-being trajectories during the COVID-19 pandemic while caring for a relative in LTC. Overall, we found little evidence of major negative effects on caregiver depressive symptoms, self-efficacy, and caregiving burden on average. Although there was substantial variability in individuals’ experiences, this variability was largely unexplained by factors one might expect, such as the type and closeness of the relationship between caregiver and care recipient, the caregivers’ satisfaction with the LTC facility’s COVID-19 policies, or the care recipient being infected with COVID-19. Caregivers’ well-being may have been more substantially influenced by factors we could not examine in detail in the present study, such as socioeconomic status and marginalization based on race or ethnicity or facility-/community-level characteristics that contributed to successful management of COVID-19 or lack thereof. Additionally, caregivers whose well-being did not appear to be negatively affected by the challenges of the pandemic may have been buoyed by utilizing coping strategies. Caregivers in the parent RCTM study shared that employing effective communication strategies and relaxation exercises helped manage the stress of caregiving challenges (Albers et al., 2023; Zmora et al, 2021). For caregivers whose well-being is negatively impacted, targeted support including a focus on strategies to help improve communication between the caregiver and center staff should be provided, particularly to caregivers whose relative’s care significantly deteriorates after the onset of a pandemic or a similar disruptive event. Caregivers whose relatives have less severe behavioral symptoms may also benefit from additional psychosocial support. However, our findings also emphasize the utility of effective coping strategies, strategies that can be taught, and the strength of caregivers’ resilience in navigating the unprecedented disruptions and threats related to the pandemic.

## Supplementary Material

igad034_suppl_Supplementary_Material

## Data Availability

Data, analytic code, and study materials are available upon request from the corresponding author. The hypotheses and analytic plan for this study were preregistered on the Open Science Framework: https://osf.io/dfsg7
