# Supplementary material for: Caring for a Relative With Dementia in Long-Term Care During the COVID-19 Pandemic: A Prospective Longitudinal Study
Source: Innov Aging. 2023 Apr 17;7(4):igad034. doi: 10.1093/geroni/igad034 (PMC10195573; doi:10.1093/geroni/igad034)
Supplement: igad034_suppl_Supplementary_Material [file igad034_suppl_supplementary_material.docx]

**Online Supplementary Material**

Table S1. *Correlations Among Descriptive Statistics and Outcomes: Depressive Symptoms*

|  | CES-D W1 | CES-D W2 | CES-D W3 | CES-D W4 | CES-D W5 | CES-D W6 | CES-D W7 |
| --- | --- | --- | --- | --- | --- | --- | --- |
| 1. Primary Caregiver | .11 | .13 | .04 | .05 | .05 | .05 | .10 |
| 2. Caregiver Female | .15 | .06 | .23* | .21* | .17 | .14 | .17 |
| 3. Caregiver Age | .001 | .02 | -.05 | -.03 | -.05 | -.08 | -.12 |
| 4. Caregiver Race/Ethnicity | .33*** | .28** | .23** | .26** | .11 | .16 | .18 |
| 5. Caregiver Married | -.18* | -.28** | -.35*** | -.16 | -.17 | -.08 | -.13 |
| 6. Caregiver Children | -.09 | -.12 | -.17 | -.04 | -.15 | -.04 | -.03 |
| 7. Caregiver Education | .09 | .16 | .12 | .008 | .14 | .08 | .23 |
| 8. Caregiver Income | -.16 | -.17 | -.19* | -.29** | -.23* | -.17 | -.16 |
| 9. Caregiver Work | .02 | .13 | .10 | .08 | .009 | <.001 | -.05 |
| 10. Care Recipient Female | -.20* | -.14 | -.09 | -.16 | -.11 | -.17 | -.15 |
| 11. Care Recipient Age | -.10 | -.19* | -.10 | -.04 | -.14 | -.13 | .01 |
| 12. Care Recipient Race/Ethnicity | .15 | .13 | .13 | .05 | .13 | .16 | .35** |
| 13. Care Recipient Married | -.009 | -.005 | -.08 | .01 | -.06 | -.05 | -.10 |
| 14. Care Recipient Children | -.08 | -.14 | -.03 | .05 | -.06 | .02 | -.008 |
| 15. Care Recipient Education | -.02 | .10 | -.06 | -.06 | -.06 | -.06 | -.02 |
| 16. Care Recipient Income | -.03 | -.08 | -.24** | -.08 | -.10 | -.04 | -.09 |
| 17. Care Recipient Medicaid | -.11 | -.11 | .08 | .11 | .01 | -.01 | .02 |

*Note*. CES-D = Center for Epidemiological Studies – Depression Scale; W = Wave.

Table S2. *Correlations Among Descriptive Statistics and Outcomes: Caregiver Self-Efficacy*

|  | CSE W1 | CSE W2 | CSE W3 | CSE W4 | CSE W5 | CSE W6 | CSE W7 |
| --- | --- | --- | --- | --- | --- | --- | --- |
| 1. Primary Caregiver | -.01 | .04 | -.10 | -.03 | -.02 | -.06 | .03 |
| 2. Caregiver Female | -.08 | -.11 | -.18 | -.01 | .01 | .001 | .26 |
| 3. Caregiver Age | .09 | -.05 | -.10 | -.18 | -.05 | -.08 | -.18 |
| 4. Caregiver Race/Ethnicity | -.17* | -.07 | -.03 | -.20* | -.19 | -.23* | -.05 |
| 5. Caregiver Married | .18* | .25** | .19* | .23* | .14 | .13 | .09 |
| 6. Caregiver Children | -.06 | .05 | .14 | .01 | .06 | .06 | .08 |
| 7. Caregiver Education | .08 | .12 | .13 | .11 | .06 | -.05 | .28 |
| 8. Caregiver Income | .11 | .04 | .31** | .38*** | .25* | .13 | .49** |
| 9. Caregiver Work | .06 | -.05 | -.08 | -.13 | -.04 | -.10 | -.18 |
| 10. Care Recipient Female | .05 | -.04 | .04 | -.07 | .02 | -.01 | .13 |
| 11. Care Recipient Age | .09 | -.01 | -.03 | -.02 | .06 | -.10 | .16 |
| 12. Care Recipient Race/Ethnicity | -.10 | .01 | -.05 | -.05 | -.06 | -.05 | -.04 |
| 13. Care Recipient Married | -.10 | -.02 | -.12 | -.17 | -.20 | -.06 | -.26 |
| 14. Care Recipient Children | -.05 | -.08 | -.05 | -.12 | -.10 | -.12 | -.07 |
| 15. Care Recipient Education | -.13 | .08 | .04 | -.02 | .08 | .05 | -.10 |
| 16. Care Recipient Income | -.02 | .27** | .18 | .08 | .05 | .15 | .001 |
| 17. Care Recipient Medicaid | .13 | -.03 | .02 | .01 | -.01 | -.01 | .07 |

*Note*. CSE = Caregiver self-efficacy; W = Wave.

Table S3. *Correlations Among Descriptive Statistics and Outcomes: Caregiver Burden*

|  | ZBI W1 | ZBI W2 | ZBI W3 | ZBI W4 | ZBI W5 | ZBI W6 | ZBI W7 |
| --- | --- | --- | --- | --- | --- | --- | --- |
| 1. Primary Caregiver | .19* | .17 | .20* | .20* | .19 | .11 | -.07 |
| 2. Caregiver Female | .32** | .31** | .33** | .23* | .22* | .15 | .11 |
| 3. Caregiver Age | -.16 | -.01 | -.07 | -.06 | -.07 | -.05 | -.20 |
| 4. Caregiver Race/Ethnicity | .19* | .17 | .13 | .15 | .06 | -.01 | .25 |
| 5. Caregiver Married | -.19* | -.14 | -.20* | -.10 | -.03 | -.11 | -.09 |
| 6. Caregiver Children | -.16 | -.17 | -.19* | -.13 | -.23* | -.17 | -.05 |
| 7. Caregiver Education | .13 | .06 | .08 | .14 | .11 | .11 | .06 |
| 8. Caregiver Income | -.05 | -.15 | -.18 | -.15 | -.13 | -.10 | -.27 |
| 9. Caregiver Work | -.04 | .05 | -.05 | -.01 | -.06 | -.03 | .05 |
| 10. Care Recipient Female | -.09 | -.11 | -.09 | .003 | -.01 | .10 | .02 |
| 11. Care Recipient Age | -.11 | -.07 | -.07 | -.003 | .03 | .10 | .20 |
| 12. Care Recipient Race/Ethnicity | .16 | .02 | .07 | -.02 | .02 | .02 | .17 |
| 13. Care Recipient Married | -.21* | -.09 | -.08 | -.06 | -.07 | -.11 | -.14 |
| 14. Care Recipient Children | -.03 | -.02 | .02 | .02 | .06 | .13 | .29 |
| 15. Care Recipient Education | .07 | .06 | .004 | -.04 | -.16 | -.17 | -.35* |
| 16. Care Recipient Income | -.05 | -.01 | -.11 | -.07 | -.09 | -.10 | -.13 |
| 17. Care Recipient Medicaid | -.20* | -.16 | -.17 | -.19* | -.08 | -.11 | .01 |

*Note*. ZBI = Zarit Burden Inventory; W = Wave.

Table S4. Analyses Comparing Participants with Seven Assessments to Participants with Fewer Assessments

|  | 7 Assessments | | | <7 Assessments | | | Comparison | |
| --- | --- | --- | --- | --- | --- | --- | --- | --- |
|  | M | SD | % | M | SD | % | *t*-test | Chi-square |
| Treatment group | - | - | 42.6 | - | - | 50.7 | - | χ^2^=.86, *df*=1, *p*=.35 |
| Race/Ethnicity | - | - | 4.9 | - | - | 1.4 | - | χ^2^=1.38, *df*=1, *p*=.24 |
| Married | - | - | 80.3 | - | - | 81.7 | - | χ^2^=.04, *df*=1, *p*=.84 |
| Income | 8.66 | 1.82 | - | 9.03 | 1.54 | - | *t*=-1.24, *df=*127, *p*=.22 | - |
| Primary Caregiver | - | - | 90.2 | - | - | 83.1 | - | χ^2^=1.39, *df*=1, *p*=.24 |
| Female | - | - | 88.5 | - | - | 83.1 | - | χ^2^=.78, *df*=1, *p*=.38 |
| Number of children | 2.07 | 1.55 | - | 1.97 | 1.54 | - | *t*=.35, *df=*130, *p*=.73 | - |
| Medicaid | - | - | 24.6 | - | - | 34.3 | - | χ^2^=1.47, *df*=1, *p*=.23 |
| Spouse | - | - | 21.3 | - | - | 35.2 | - | χ^2^=3.09, *df*=1, *p*=.08 |
| Memory Impairment | 3.22 | .70 | - | 3.33 | .85 | - | *t*=-.84, *df=*130, *p*=.40 | - |
| RMBPC-Frequency | 1.83 | .56 | - | 1.82 | .66 | - | *t*=.11, *df=*130, *p*=.92 | - |
| RMBPC-Severity | 1.28 | .58 | - | 1.38 | .80 | - | *t*=-.77, *df=*128, *p*=.44 | - |
| Relationship Closeness | 3.07 | .51 | - | 3.71 | .74 | - | *t*=-5.76, *df=*130, *p*<.001 | - |
| Staff Support | 2.31 | .38 | - | 2.45 | .41 | - | *t*=-2.02, *df=*130, *p*=.046 | - |
| Satisfaction with LTC | 3.95 | .89 | - | 4.07 | .66 | - | *t*=-.84, *df=*114, *p*=.40 | - |
| Bereaved by W7 | - | - | 29.5 | - | - | 35.2 | - | χ^2^=.49, *df*=1, *p*=.49 |
| Baseline Depressive Symptoms | .72 | .56 | - | .68 | .49 | - | *t*=.43, *df=*130, *p*=.67 | - |
| Baseline Self-Efficacy | 3.17 | .58 | - | 3.50 | .81 | - | *t*=-2.71, *df=*128, *p*=.008 | - |
| Baseline Burden | 1.93 | .79 | - | 1.74 | .82 | - | *t*=1.37, *df=*130, *p*=.17 | - |

*Note*. RMBPC = Revised Memory and Behavior Problems Checklist; LTC = long-term care; W = Wave. Memory Impairment, RMBPC-Frequency, RMBPC-Severity, Relationship Closeness, and Staff Support were calculated as the mean of all available assessments prior to pandemic onset.
